# Optimization of spray‐drying process to manufacture green tea powder and its characters

**DOI:** 10.1002/fsn3.2597

**Published:** 2021-10-20

**Authors:** Dong Thi Anh Dao, Hoang Van Thanh, Do Viet Ha, Vuong Duc Nguyen

**Affiliations:** ^1^ Department of Food Technology Faculty of Chemical Engineering Ho Chi Minh City University of Technology (HCMUT) Ho Chi Minh City Vietnam; ^2^ Vietnam National University Ho Chi Minh City Ho Chi Minh City Vietnam; ^3^ Center of Experimental Practice Ho Chi Minh City University of Food Industry Ho Chi Minh City Vietnam; ^4^ Management Board of Agricultural Hi‐Tech Park The People’s Committee of Ho Chi Minh City Ho Chi Minh City Vietnam; ^5^ Institute of Biotechnology and Food Technology Industrial University of Ho Chi Minh City 12 Nguyen Van Bao, Ward 4, Go Vap District Ho Chi Minh City Vietnam

**Keywords:** EGCG, green tea, optimization, response surface methodology, spray‐drying, TPC

## Abstract

Tea leaves (*Camellia sinensis*) have many health benefits due to their abundance of polyphenols with antioxidant activity, most notably epigallocatechin‐3‐gallate (EGCG). To protect those bioactive compounds, the spray‐drying technique of green tea‐extracted solution is conducted because of encapsulating. This study aimed to optimize the spray‐drying condition using the response surface methodology (RSM) with respect to the maximal polyphenol content of the product. Furthermore, the characterizations of resulting powder were determined. The results showed that optimal spray‐drying temperature, input flow rate, and whey protein isolate (WPI) content were evaluated at 136℃, 6.8 rpm, and 10.3% of dry basis, respectively. The obtained green tea powder products, which got from optimal spray‐drying process, achieved total polyphenol content (TPC), EGCG, and caffeine content of 322.06 mg GAE/g, 11.4%, and 2.8% of dry basis, respectively. This result revealed the feasibility of green tea leaves to produce tea powder rich in EGCG and polyphenols by spray‐drying technique, potentially contributing to the diversification of tea products.

## Graphical abstract

The spray‐drying process to make tea powder from the extract was optimized by RSM with response of total polyphenol. The tea powder product shows attractive characterization of remained total polyphenol, chlorophyll, EGCG, and caffeine.

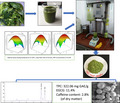

## 1 INTRODUCTION

An infusion prepared with dried tea leaves is one of the most popular beverages in Asian countries, and its benefits on human health have been extensively studied. It has been shown that antioxidants such as phenols and flavonoids present in the leaf are responsible for a wide range of anti‐aging and beneficial effects (Cabrera et al., 2006; Unno et al., 2007). Besides, green tea is also a plant material that could aid in the treatment of diseases that involves the gastrointestinal system such as obesity, type II diabetes, and reduce the risk for cancer, liver, and coronary heart diseases (Koo & Cho, 2004). Therefore, exhaustive extraction of bioactive compounds from tea secondary resources and tea wastes might play an important role in enhancing the valorization of tea trees.

While young tea leaves have been traditionally used to prepare infusion beverages, old tea leaves are a common ingredient in the extraction of bioactive substances. Among the beneficial ingredients in tea leaves, polyphenols are the most important group of bioactive compounds, accounting for approximately 30% of the dry matter content of fresh tea leaves and comprise four main groups including phenolics acid, stilbene, flavonoids, and Lignin. Tea flavonoids, often called catechins, are further classified into four main types including epicatechin (EC), epigallocatechin (EGC), epicatechin‐3‐gallate (ECG), and epigallocatechin gallate (EGCG) (Sano et al., 2001). Of which, EGCG has the ability to prevent oxidation and prevent cancer (Du et al., 2012).

In Vietnam, tea tree (*Camellia sinensis*) is grown mainly in the Central Highlands with a production area of nearly 130 thousand hectares, giving the annual total output of dried tea leaves ranging from 140 to 150 thousand tons. Despite such high production of tea material, consumer demands for diversified tea products have called for the development of advanced techniques that allow the production of tea powder with improved solubility and stability. Dehydration through spray‐drying is one technique widely used in the food industry, and under optimal processing conditions, it has been proven to be an effective method to obtain various products. Transformation of product into a dry particulate form results in much‐reduced volume and longer shelf life (Cano‐Chauca et al., 2005). The process carries industrial implications due to quick drying time and is particularly suitable to encapsulate materials that contain thermally sensitive compounds (Mishraa et al., 2014). Spray‐drying has been successfully applied for polyphenol stability in plant foods such as bayberry polyphenols (Fang & Bhandari, 2011); micro‐encapsulation of polyphenol bioactive (Sun‐Waterhouse et al., 2013); pomegranate (*Punica granatum*) (Robert et al., 2010); and blueberry polyphenol (Hoskin et al., 2018). In this study, we utilized spray‐drying technique to produce powdered green tea extract and optimize the process with respect to maximal polyphenol and EGCG content. Various process parameters including drying temperature, input flow rate, and whey protein isolate content were taken into optimization model. The results are expected to aid in further development of manufacturing process of soluble powder green tea at a larger scale.

## 2 MATERIALS AND METHODS

### 2.1 Materials

Fresh tea leaves were collected from Cau Dat tea garden, in Xuan Truong commune, Da Lat City, Lam Dong Province, Vietnam. Tea leaves were stored in PE bags (20*40cm) with perforations (hole diameter of 1 cm, 10 holes/bag) at 15℃. Materials were stored for a maximum time of 4 days before being used in experiments.

Drying additive, Hilmar 9,410 instantized whey protein isolate (WPI), was obtained from Hilmar Ingredients company (California, US), that is with the following components: protein content of 89%, moisture content of 4.7%, pH 6.2÷7.0, lactose content of 0.1%, fat content of 1.3%, and ash of 2.7%.

### 2.2 Extract preparation

Tea leaves were washed and allowed to dry naturally. In each experiment, 200g of fresh tea leaves was blanched at 90℃ for 30÷45s ( Tran et al., 2018), then was withdrawn, and cooled for 10÷15 min at 0–5℃ before being allowed to do further experiments. Blanched tea leaves were then ground by using a blender (Phillips HR2096/00) to the size smaller than 0.5 mm and extracted with water under assistance of pectinase and cellulase enzyme system. Extraction conditions included material/water ratio of 1/4 (g/mL), pectinase/cellulase ratio of 1/2 (v/v), enzyme mixture content by dry basis of raw material of 4% (v/w), extraction time of 30 min, and extraction temperature of 55℃, for the 1st cycle. Next, the 2nd cycle of extraction was conducted by adding 98% ethanol solvent (v/v) to achieve 30% (v/v) ethanol with duration time of 10 min. Extracted mixture was filtered through a 0.2 mm sieve, and the permeate was mixed with WPI to investigate the spray‐drying process.

### 2.3 Spray‐drying process

Spray‐drying is well‐established method for converting liquid feed materials into a dry powder form with microencapsulated. During the spray‐drying process, the liquid feed is first atomized and contacted with hot air; evaporation takes place to yield dried particles, which are subsequently separated from the air stream by a variety of methods (Anandharamakrishnan et al., 2007). The instrument used in this study was SD06, Labplant, UK with the following specifications:

Heating capacity: 3 kW
Evaporation capacity: 1,000÷1,500 mL/h
Dry matter content of input flow: 6.3%
Air throughput capacity: 15÷30 m^3^/h
Maximum inlet temperature: 250℃
Air Pressure: 2 m^3^/h at 2 bar ÷ 1.7 m^3^/h at 4 bar

### 2.4 Determination of total polyphenol content

Total polyphenol content was determined according to the Folin–Ciocalteu method described by Fu et al. (2011). Dilute the solution after enzyme extraction. Then, 0.5 mL of the diluted solution was taken into a test tube, which was added with 2.5 mL of ten‐fold diluted Folin–Ciocalteu solution. The tube was vortexed and allowed to react for 4 min. Afterward, 2.0 mL of 7.5% Na_2_CO_3_ solution was added into the tube, followed by shaking. The solution was left in the dark at room temperature for 2 h before being spectrophotometrically measured by optical absorbance (PhotoLab 6100 Vis, WTW, Germany) at the wavelength of 760 nm. Gallic acid was used as the standard. The same procedure was carried out to prepare the blank sample, except that 0.5 mL of sample was replaced with the same volume of distilled water. The total polyphenol content is calculated by the formula (1).

(1) $$X\left(\text{mg}\;\text{GAE}/g\;\text{dry}\;\text{basis}\right)=\frac{x*V*f}{1000*m*(1‐w)}$$

where: X: total polyphenol content per mg gallic acid per gram of dry basis (mg GAE/g dry basis); x: concentration of mg gallic acid determined by standard curve (ppm); V: volume of extract solution from m (g) of sample (ml); f: dilution factor; 1000: conversion factor to gram; m: mass of sample to be extracted (g); w: humidity (%).

### 2.5 Determination of chlorophyll content

Chlorophyll content of extracted powder was determined as previously described (Lichtenthaler & Buschmann, 2001). The solution was first diluted to an appropriate concentration. Then, the optical absorbance of the solution was measured by spectrophotometer (PhotoLab 6100 Vis, WTW, Germany) at the wavelength of 664 and 648 nm. The blank sample was prepared identically with sample being replaced with distilled water. Content of chlorophyll a, chlorophyll b, and total chlorophyll is calculated by the formula (2), 3), and (4), respectively. Chlorophyll content is calculated by the formula (5).

(2) $$\text{Chlorophyll}\;a\;\left(\mu g/\text{ml}\right)=12.25*A^{664}‐2.55*A^{648}$$

(3) $$\text{Chlorophyll}\;b\;\left(\mu g/\text{ml}\right)=20.31*A^{648}‐4.91*A^{664}$$

(4) $$\text{Total chlorophyll}\;b\;\left(\mu g/\text{ml}\right)=7.34*A^{664}‐17.76*A^{648}$$

(5) $$\text{Chlorophyll}\;\text{content}\;\text{of}\;\text{powder}\;\left(\text{mg}/g\;\text{dry}\;\text{basis}\right)=\frac{X*V*f}{1000*m*(1‐w)}$$

where: A^664^ and A^648^: Absorbance of sample at wavelength of 664 and 648 nm, respectively; X: total chlorophyll (μg/mL); V: volume of extract solution from m (g) of sample (mL); f: dilution factor; 1000: conversion factor from μg to mg; m: mass of sample to be extracted (g); w: humidity of sample (%).

### 2.6 Determination of EGCG and caffeine content by HPLC method

The EGCG and caffeine content in green tea extracts and green tea powder products were quantified by HPLC (High‐Performance Liquid Chromatography) (Lu & Chen, 2008) by using Agilent 1260 HPLC system (USA) equipped with a DAD detector (Detector Diod Array) and Agilent's LiChrospher® 100 RP‐18 (5 µm – 250 × 3.0 mm) column. The measurement wavelength was 280 nm, sample loading volume was 20 µL, and flow rate was 1 mL/min. The instrument operated at isocratic with the mobile phase of Acetonitrile‐Water solvent system at 15:85 (v/v). EGCG standards purchased from Sigma (USA) with a minimum content of 98% were used to prepare a series of standard solutions. The sample was extracted with the solvent MeOH: H_2_O at 7:3 (v/v) at 70℃, 30 min by ultrasound, removing impurities, and was determined by HPLC method.

### 2.7 Size of tea powder determination

Morphology of the microencapsulated tea extract was observed via Scanning Electron Microscopy (*SEM*) technique at the magnification level of 5,000 and 10,000. The measurement was carried out at Key Laboratory of Polymer Materials and Composites, Ho Chi Minh City University of Technology (HCMUT).

### 2.8 Design of optimal experiment

Experimental parameters are arranged according to the method of satisfying the structural surface with center. Three experimental conditions, including drying temperature (X_1_), input flow rate (X_2_), and WPI content (X_3_), which mainly affect total polyphenol content (TPC) of green tea powder, were taken into optimization model based on response surface methodology. From the preliminary experiments on impact of individual factors on the outcome, the center values of the elements were determined and subsequently used in establishing sets of parameters, which were then experimentally attempted to generate data for model estimation. To examine the optimal area for the three factors, each factor takes one out of 5 levels: ‐α, −1, 0, +1, and +α, where 0 is the center value and α = 1.68. The number of experiments is determined as follows: *N* = 2^3^+ 2*3 + 6 = 20. The design of experiment layout of rotatable CCD was presented in Table 1. The regression takes the following Equation 6, where Y represents polyphenol content and is described as a function of three independent factors (X_1_, X_2_, X_3_).

(6) $$Y=b_{0}+b_{1}X_{1}+b_{2}X_{2}+b_{3}X_{3}+b_{11}X_{1}^{2}+b_{22}X_{2}^{2}+b_{33}X_{3}^{2}+b_{12}X_{1}X_{2}+b_{23}X_{2}X_{3}+b_{13}X_{1}X_{3}$$

**TABLE 1:** Design of experiment layout of rotatable CCD with three independent factors and the observed results of a response

Run	Independent factors			Response total polyphenol (mg GAE/g dry basis)
	Spray‐drying temperature (°C)	Input flow rate (rpm)	WPI content (%)	
1	120	6	5	224.39
2	160	6	5	198.3
3	120	8	5	207.53
4	160	8	5	192.55
5	120	6	15	235.13
6	160	6	15	205.78
7	120	8	15	203.03
8	160	8	15	179.9
9	106.36	7	10	288.13
10	173.64	7	10	246.07
11	140	5.318	10	287.4
12	140	8.682	10	265.9
13	140	7	1.59	292.04
14	140	7	18.41	301.89
15	140	7	10	287.45
16	140	7	10	324.73
17	140	7	10	319.68
18	140	7	10	320.3
19	140	7	10	294.4
20	140	7	10	304.95

In which, b_1_, b_2_, and b_3_ represent the coefficients of 1st order; b_11_, b_22_, and b_33_ are the coefficients of 2nd order; b_12_, b_23_, and b_13_ are the interaction coefficients of each interaction pair of factors X_1_, X_2_, and X_3_.

After obtaining via predictive calculations, set of optimal parameters was validated by conducting experiments.

### 2.9 Data processing

All the experiments in this study were repeated in triplicate. The experiment for optimization was designed and analyzed by using MODDE 5 software.

## 3 RESULTS AND DISCUSSION

### 3.1 Effect of spray‐drying temperature on polyphenol content

**FIGURE 1:**
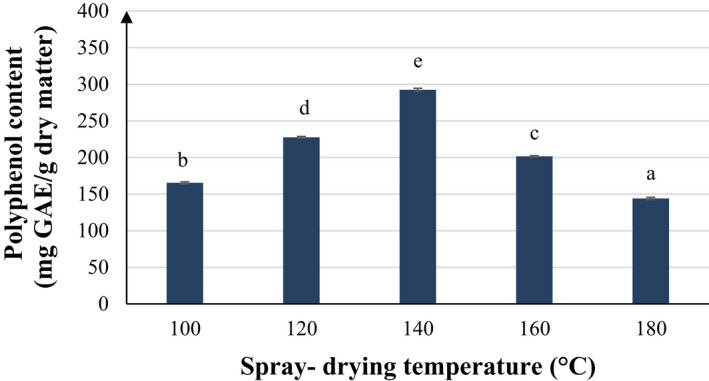
Effect of spray‐drying temperature on polyphenol content after spray‐drying. The characters a, b, c, d, and e present the significant difference of each average polyphenol content at confident interval of 95%

Polyphenols are sensitive to elevated temperature, which also causes discoloration of the powder product. However, very low drying temperature might result in higher moisture content of the product, leading to product adhesion in the drying chamber and in turn reduced recovery efficiency. In this study, the inlet drying temperature was surveyed at 5 levels ranging from 100 to 180℃. Other parameters were fixed at input flow rate of 6 rpm (5.4 mL/min) and WPI content of 5% of the carrier dry matter. When the temperature increased from 100 to 140℃, the TPC also increased from 165.44 to 292.38 mg GAE/g dry basis. The TPC got peak of 292.38 ± 2.14 mg GAE/g dry basis at temperature of 140℃. Rising the temperature over 140℃ led to strong decline in TPC (Figure 1). During spray‐drying process, higher temperature is associated with higher rate of heat transfer to the material particles, accelerating the evaporation of water and thus generating more steam (Rodríguez‐Hernández et al., 2005). The reverse effect of high temperature on polyphenol content was elaborated by Gupta et al. (2011) proposing that heat might structurally alter polyphenol compounds and bind polyphenols with other compounds, leading to reduced total polyphenol content. Another explanation was provided by Georgetti et al. (2008) which suggested that isoflavone degradation could be linked with heat‐induced oxidation or decomposition of thermally sensitive substances and that the spray‐drying process could reduce the quantities of volatiles in the feed material (Georgettia et al., 2008).

The inverse relationship between inlet air temperature and moisture content in the product was also observed in the study Ferrari et al. (2012). Lower moisture content could in turn impair stability of the powder in later storage. This is further elaborated by Tonon et al. (2008) speculating that lower moisture powders are more capable to absorb ambient moisture due to higher differences in water concentration between powder and the storage environment.

On the other hand, sensorial examination of the powder product indicated that characteristically green color and aroma were less pronounced in the powder product obtained at high drying temperature. This observation is in line with a result of a previous study where spray‐dried mixture of fermented mixed carrot and watermelon juices exhibited less intensities in red and orange color as the inlet temperature was elevated from 120 to 160℃ (Mestry et al., 2011).

### 3.2 Effect of input flow to the polyphenol content

**FIGURE 2:**
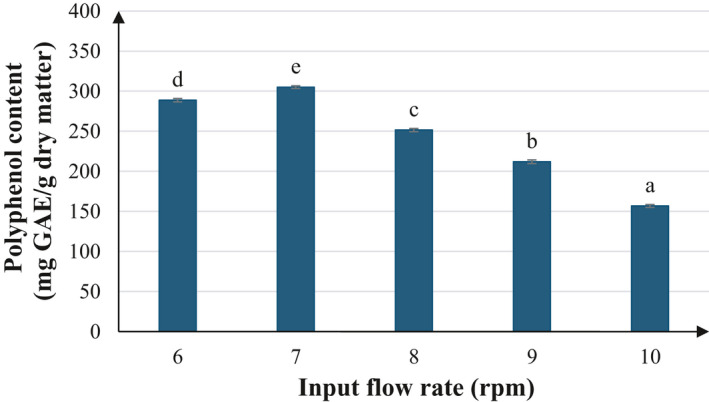
Effect of input flow rate of the polyphenol content after spray drying. The characters a, b, c, d, and e present the significant difference of each average polyphenol content at confident interval of 95%

From the experimental results, it is revealed that the flow rate exerts clear effect on the polyphenol content. As the flow rate increased until certain threshold, the TPC was also improved proportionally (Figure 2). To be specific, as the input flow rate rose from 6 to 7 rpm, TPC was climbed from 288.82 to 305.15 mg GAE/g dry basis. Afterward, continuing to increase the flow rate to very high level gradually reduced the TPC. The comparatively lower TPC at lower feed flow rate could be explained by the drying time that is longer than required to remove the moisture, causing the sample to be exposed to longer heat in the drying chamber and in turn leading to decreased polyphenol content. On the contrary, at very high flow rates, the moisture in the feed and the product tends to be higher due to insufficient drying time. This impairs recovery efficiency and thus reduces total polyphenol content of the product.

Other studies suggested that with higher pumping rates, the heat of the hot air agent cannot be transferred to the material droplets in the drying chamber; this results in a reduced water separation and increased moisture content of the product (Fu et al., 2011). According to Hong and Choi (2007), the moisture content of powders was increased with increasing pump rate. Tonon et al. (2008) also reported the negative influence of feed flow rate on drying yield in acai (Euterpe oleraceae Mart.) powder (Tonon et al., 2008). This was explained by the fact that more rapid flow rate may hamper heat and mass transfer and cause dripping of excess, non‐atomized feed through the nozzle, which in turn reduces drying yield.

### 3.3 Effect of the proportion of added WPI on polyphenol content

**FIGURE 3:**
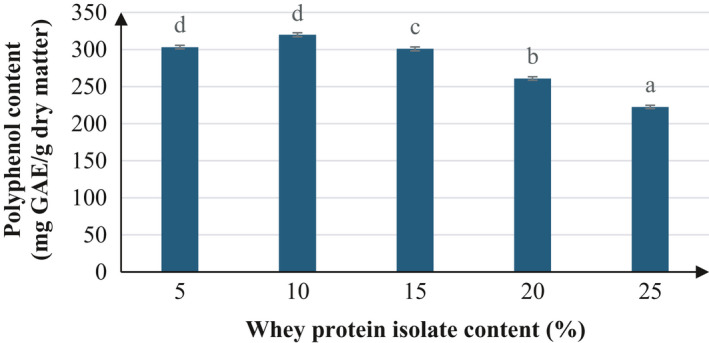
Effects of whey protein isolate content on polyphenol content after spray‐drying. The characters a, b, c, d, and e present the significant difference of each average polyphenol content at confident interval of 95%

The ratio of added WPI based on the dry matter of the extract was allowed to vary from 5% to 25% dry basis, and its impact of total polyphenol was presented in Figure 3. There is a significant difference in the amount of polyphenol recovered between the investigated concentrations (*p* < 0.05). The polyphenol content increased with an increase in WPI additions from 5% to 10%, at which point the TPC reached the highest value of 319.82 mg GAE/g dry basis. However, when the WPI supplementation rate exceeded 10%, the TPC tended to decrease gradually. Higher quantity of WPI addition is closely linked with improved powder recovery efficiency.

Qilong Shi et al. suggested that the adhesion property (stickiness) between the particles and the wall of the drying chamber are very important in spray‐drying. Wall deposits will be produced if the droplet surface is dominated by low molecular sugars (Bhandari & Howes, 2005; Shi et al., 2013). According to Bhesh Bhandari et al., powder may deposit on walls, or a blockage may occur in pipes or cyclones. Moreover, after spray‐ drying, powder has the heat exchanger with the glass cycle (cycle of spray‐drying machine) very slow; so the output powder product with high temperature will absorb moisture, leading to an increase in moisture content (Bhandari & Howes, 2005).

However, when WPI is added to the input solution, the protein molecules of WPI migrate to the air‐water interface of the solution. The result of this is to overcome the coalescence of the droplets and adhesion of the particles in the drying chamber of the spray dryer (Adhikari et al., 2007; Shi et al., 2013). This leads to an increase in the recovery efficiency of drying powder, and polyphenol is also covered. However, when the content of WPI increases, the polyphenol content will be reduced significantly (Pang et al., 2014). Because of the higher protein concentration in the feed due to higher denaturation of protein, it can cause blockage in pipes or cyclones. The equipment must be repaired, the drying process is interrupted, polyphenols in the input fluid will be lost, so the TPC value will be decreased.

Moreover, excessive addition of WPI can increase the viscosity of the spray‐drying fluid, resulting in larger droplet size, and subsequently obstructing the mass transfer of moisture from the inside to the outside of the droplet. This necessitates longer drying time and possibly introduces nozzle blockage, resulting in reduced recovery efficiency and polyphenol content due to product adhesion in the chamber.

Similar to the effect of the drying temperature, the moisture content of the powder also decreases as the WPI content increases and thus affects the powder's stability during storage.

### 3.4 Optimization of the spray‐drying process

The obtained data of response (Table 1) were then used to estimate the response surface methodology (RSM) quadratic function. Based on the results of ANOVA, non‐significant affected elements were removed from the equation, giving the reduced model that describes the relationship between polyphenol content (Y) and experimental parameters as follows.

$$Y=310.924‐29.998\;X_{1}^{2}‐26.623\;X_{2}^{2}$$

**FIGURE 4:**
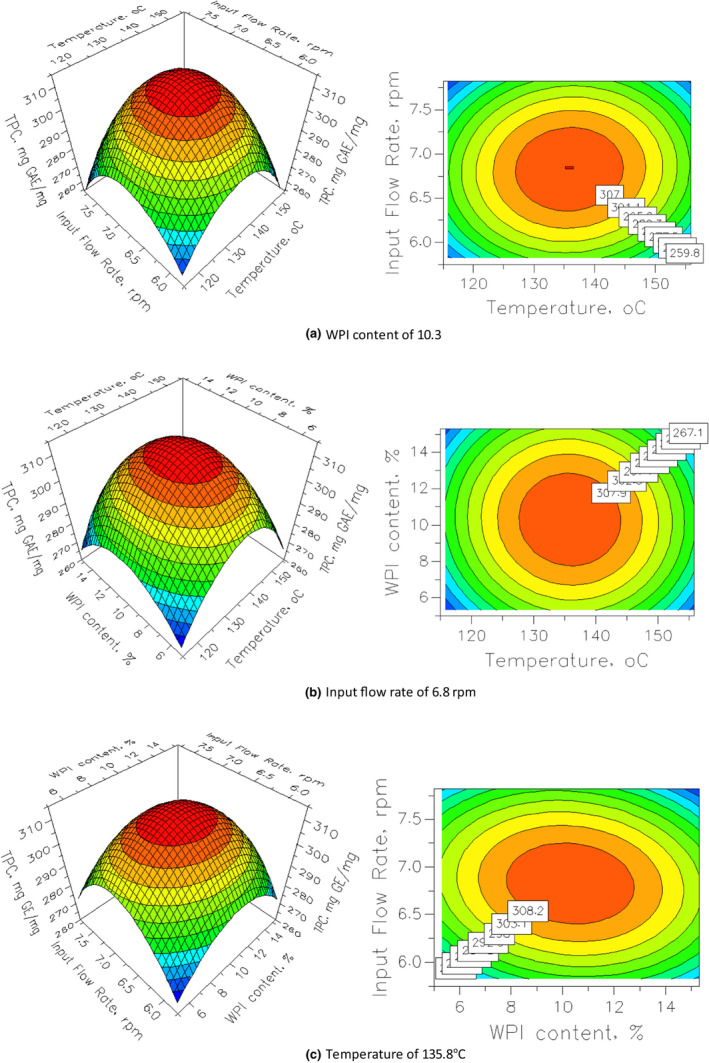
Response surface and contour plots by factors of input flow rate versus. WPI content (a), input flow rate versus. spray‐drying temperature (b), and spray‐drying temperature versus. WPI content (c) at the stationary point

The relationship between the spray‐drying conditions and the polyphenol content was depicted in Figure 4. In each surface, one variable was kept at its optimal values and the remainder was allowed to vary. The hill‐like shape of response surfaces implies that all factors had a significant effect on TPC. In addition, the pairwise interaction of two variables on the response was seemed to follow a similar pattern where increased flow rate, WPI content, or drying temperature leads to reduced polyphenol content. However, the trend only holds to a certain parameter threshold after which the reverse pattern began to manifest. At the optimal conditions of spray‐drying process, including spray‐drying temperature of 135.8℃, input flow rate of 6.8 rpm and the WPI concentration of 10.3% dry basic of input flow, the TPC of output tea powder obtained 312.96 mg GAE/g dry basis of tea powder product.

To verify this optimal condition of three parameters, the confirmatory experiment was performed at these conditions of spray‐drying temperature of 136℃, input flow rate of 6.8 rpm, and WPI concentration of 10.3%. The experiments were conducted parallel 3 times. The experimental results showed the TPC of 322.06 ± 0.05 mg GAE/g dry basis of tea powder product, which is in line with predicted response, suggesting that the regression equation is suitable to describe the outcome of the spray‐drying process.

**FIGURE 5:**
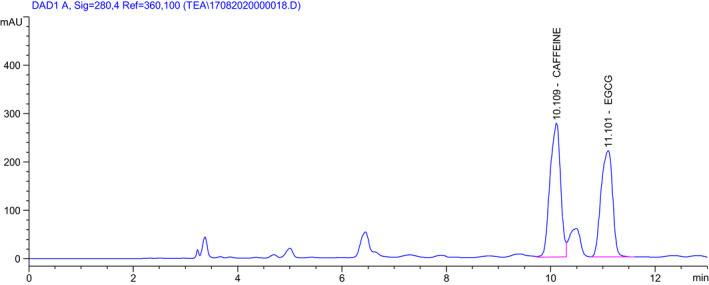
HPLC chromatography of EGCG and caffeine in tea powder after spray‐drying at optimal condition

The EGCG and caffeine content in the tea powder obtained from the optimal spray‐drying condition were quantitatively determined by HPLC–DAD (Figure 5). The EGCG and caffeine content were determined of 11.4% and 2.8% dry basis powder product respectively.

### 3.5 Particle size

**FIGURE 6:**
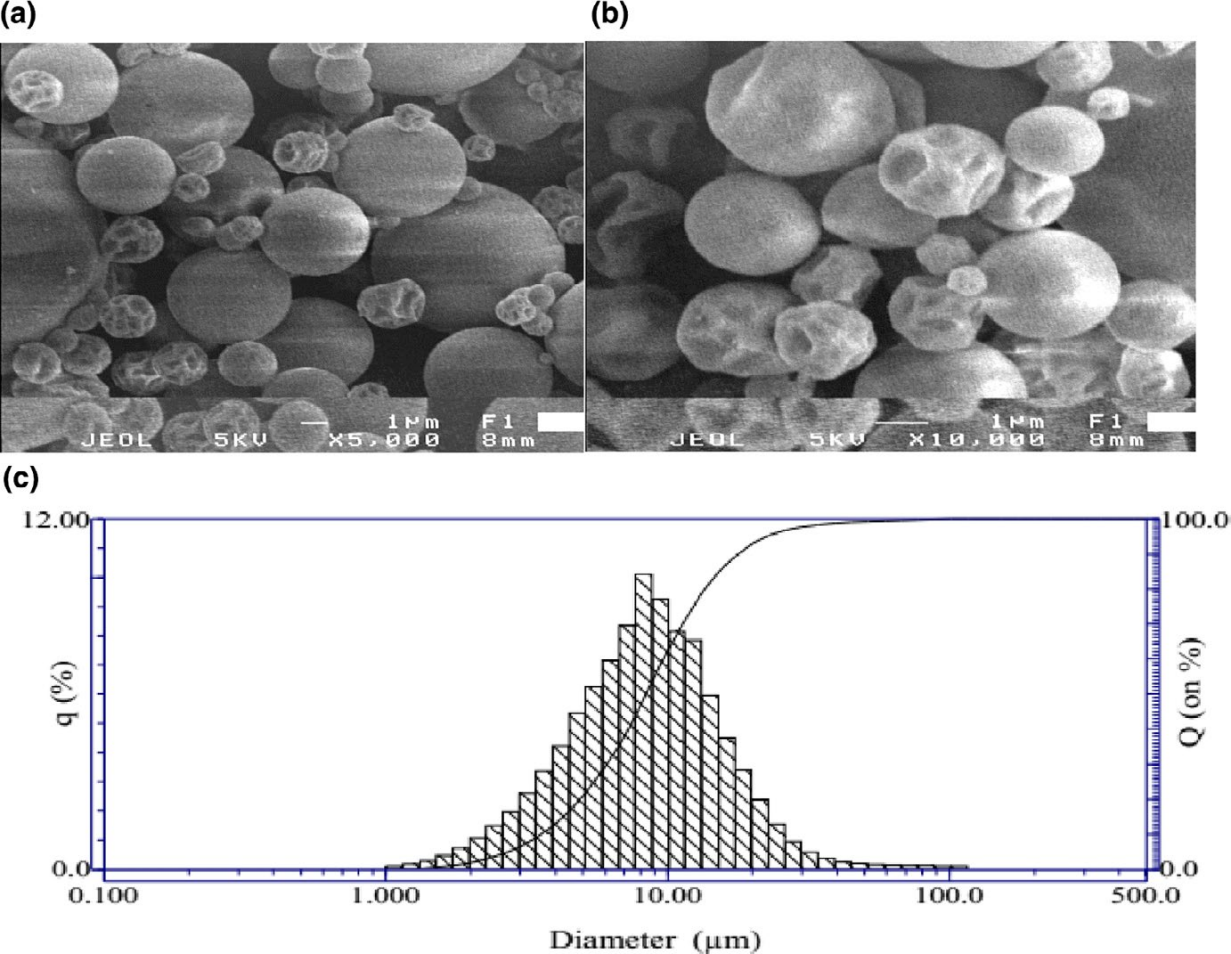
*SEM* micrographic of spray‐dried tea powder at 5,000 (a) and 10,000 magnification (b), and its size distribution (c)

Distribution of particle size largely determines characteristics such as solubility, agglomeration, and structure of the powder products. Morphology of the microencapsulated tea extract was observed via Scanning Electron Microscopy (*SEM*) technique at the magnification level of 5,000 and 10,000 and presented in Figure 6a,b, respectively. Visually, the resulting powder particles exhibited relatively fine structure with no apparent cracks or fissures. Some particles had structural deformities on the surface, possibly because of heat, friction, rapid water evaporation, or insufficient concentration for complete encapsulation of tea extract. The shriveled and concave external surface is characteristic of all spray‐dried powders (Anandharamakrishnan et al., 2007; Ezhilarasi et al., 2014). This is possibly due to uneven shrinkage and quick hardening of smaller droplets and low moisture content during the falling rate period. Figure 6c showed the diameter size distribution of the powder particles. It showed that the powder particle size was distributed in a wide range from 1.1 to 22.5 μm. Approximately 55% of the measured powder particles had their size fell within the 2.7–9.7 μm range, averaged at around 8.3 µm.

### 3.6 Chlorophyll loss

At the optimal condition, the results showed that chlorophyll content remained in the tea extract solution and in tea powder of around 93% and 87%, respectively. The chlorophyll degradation in the sample after processed could be explained by contacting with temperature and relative humidity during drying (Roshanak et al., 2016). After investigating the effect of seven different drying treatments (sun, shade, oven 60℃, oven 80℃, oven 100℃, microwave, and freeze‐drying), Roshanak et al. (2016) have revealed that the chlorophyll content which was highest remained in the freeze‐drying compared with the other.

## 4 CONCLUSION

The optimization of 3 main parameters of spray‐drying process from fresh tea leaves extraction was performed according to RSM. The response surface model was considered to be selected. The stationary point was determined as TPC maximal and accept after being checked by three times in real experiment. At the optimal condition, the resulted green tea powder was rich in TPC and EGCG as well. They were determined of 322.06 mg GAE/g and 11.4% dry basis, respectively. This result demonstrates the feasibility of green tea leaves to produce tea powder rich in EGCG and polyphenols by spray‐drying technique, potentially contributing to the diversification of tea products.

## CONFLICTS OF INTEREST

The authors declare no conflict of interest.

## AUTHOR CONTRIBUTIONS

**Dong Thi Anh Dao:** Conceptualization (equal); Investigation (equal); Methodology (equal); Supervision (equal); Writing‐review & editing (equal). **Hoang Van Thanh:** Data curation (equal); Investigation (equal); Writing‐original draft (equal). **Do Viet Ha:** Conceptualization (equal); Investigation. **Vuong Duc Nguyen:** Conceptualization (equal); Investigation; Software (equal); Writing‐review & editing (equal).

## DATA AVAILABILITY STATEMENT

Data available on request from the authors.
